# CD4-CD8-αβ and γδ T Cells Display Inflammatory and Regulatory Potentials during Human Tuberculosis

**DOI:** 10.1371/journal.pone.0050923

**Published:** 2012-12-11

**Authors:** Melina B. Pinheiro, Lis R. Antonelli, Renato Sathler-Avelar, Danielle M. Vitelli-Avelar, Silvana Spindola-de-Miranda, Tânia M. P. D. Guimarães, Andrea Teixeira-Carvalho, Olindo A. Martins-Filho, Vicente P. C. P. Toledo

**Affiliations:** 1 Departamento de Análises Clínicas e Toxicológicas, Faculdade de Farmácia, Universidade Federal de Minas Gerais, Belo Horizonte, Minas Gerais, Brasil; 2 Laboratório de Imunopatologia, Centro de Pesquisas René Rachou, Fundação Oswaldo Cruz, Belo Horizonte, Minas Gerais, Brasil; 3 Laboratório de Biomarcadores de Diagnóstico e Monitoração, Centro de Pesquisas René Rachou, Fundação Oswaldo Cruz, Belo Horizonte, Minas Gerais, Brasil; 4 Departamento de Clínica Médica, Universidade Federal de Minas Gerais, Belo Horizonte, Minas Gerais, Brasil; University of Pittsburgh, United States of America

## Abstract

T-cells play an important role controlling immunity against pathogens and therefore influence the outcome of human diseases. Although most T-lymphocytes co-express either CD4 or CD8, a smaller T-cell subset found the in the human peripheral blood that expresses the αβ or γδ T-cell-receptor (TCR) lacks the CD4 and CD8 co-receptors. These double negative (DN) T-cells have been shown to display important immunological functions in human diseases. To better understand the role of DN T-cells in human *Mycobacterium tuberculosis*, we have characterized their frequency, activation and cytokine profile in a well-defined group of tuberculosis patients, categorized as severe and non-severe based on their clinical status. Our data showed that whereas high frequency of αβ DN T-cells observed in *M. tuberculosis*-infected patients are associated with disease severity, decreased proportion of γδ DN T-cells are found in patients with severe tuberculosis. Together with activation of CD4^+^ and CD8^+^ T-cells, higher frequencies of both αβ and γδ DN T-cells from tuberculosis patients also express the chronic activation marker HLA-DR. However, the expression of CD69, an early activation marker, is selectively observed in DN T-cells. Interestingly, while αβ and γδ DN T-cells from patients with non-severe tuberculosis display a pro-inflammatory cytokine profile, characterized by enhanced IFN-γ, the γδ DN T-cells from patients with severe disease express a modulatory profile exemplified by enhanced interleukin-10 production. Overall, our findings suggest that αβ and γδ DN T-cell present disparate immunoregulatory potentials and seems to contribute to the development/maintenance of distinct clinical aspects of TB, as part of the complex immunological network triggered by the TB infection.

## Introduction

A small proportion of T lymphocytes does not express either CD4 or CD8 and can be named DN T-cells. Studies have been shown that even this minority population can be heterogeneous and several other subpopulations can be found. Thus, within the DN T lymphocyte population, cells expressing γδ or αβ TCR can be defined. γδ and αβ T-cells display distinct characteristics: recognize antigens with diverse constitution, differently processed and presented in distinct context, and are located in distinct sites in the host. αβ DN T-cells in humans have been identified as having both regulatory function and inflammatory effects associated with autoimmune disorders such as lupus and rheumatoid arthritis [1], [2], [3], [4], [5]. The alteration in the proportions of this subpopulation has also been demonstrated in infectious diseases such as leishmaniasis and Chagas, and its protective role has been described in mycobacterial infection [6], [7], [8].

The role of γδ T-cells during *M. tuberculosis* infection was first described in 1989 [9]. Similar to the αβ DN T-cell subpopulation, γδ T-cells respond to *M. tuberculosis* antigens independently of major histocompatibility complex class II recognition [9], [10]. The latter cell subpopulation was described to preferentially accumulate in inflammatory lesions and in necrotic areas of tuberculous lymphadenitis. γδ T-cells when stimulated with *M. tuberculosis* can develop cytolytic effects and produce cytokines. Most of γδ clones and also primary cells from healthy tuberculin-positive donors in response to *M. tuberculosis*-infected monocytes produce interferon-gamma (IFN-γ), but tumor necrosis factor alpha (TNF-α) can also be detected in response to phosphoantigens [11], [12]. On the contrary, in other infectious diseases, γδ T-cells were also associated with a regulatory profile exemplified by interleukin-10 production [6], [7].

Immunity against *M. tuberculosis* is cell mediated. *M. tuberculosis* resides inside macrophages, which employs a number of defense strategies against the pathogen. CD4^+^ and CD8^+^ T lymphocytes have been shown to be the major sources of IFN-γ in *M. tuberculosis* infection [13]. Moreover, interleukin-10 (IL-10) can be produced along with IFN-γ by the same T cell clones, modulating their antigen-specific proliferation and cytokine production [14], [15]. Despite that, in setting were the function of CD4^+^ and CD8^+^ T is compromised, e.g. during HIV/AIDS, other minor sources of IFN-γ might be required. Indeed, IFN-γ producing natural killer (NK) cells regulate resistance and granulocyte function during *M. tuberculosis* in mice lacking T-cells [16]. Since it has been demonstrated in several infectious and noninfectious diseases that DN T-cells are able to produce cytokines, known to be important for *M. tuberculosis* control, a detailed study of the activation state and cytokine profiles of both αβ and γδ DN T-cells in well-defined groups of tuberculosis patients was performed aiming to better understand the role that these subpopulations may have in the human immune response to *M. tuberculosis*.

## Materials and Methods

### Study population

Patients diagnosed with lung tuberculosis were selected at the Ambulatory of Reference Centers for Tuberculosis in the city of Belo Horizonte from July 2008 to September 2009. Patients, who agreed to participate in the study, answered the socioeconomic questionnaire and signed an Informed Written Consent. This study was carried out in full accordance with all International and Brazilian accepted guidelines and was approved by the Ethics Committee at the *Universidade Federal de Minas Gerais*, *COEP*, under the registration number ETIC 095/08.

Patients were classified in relation to the severity of pulmonary TB as described by AL-Moamary and co-workers in which two doctors and a radiologist to evaluate the thorax radiogram (RX) are required [17]. The severity of the disease is based on the extent of lung affected. Each lung is classified in three zones: upper, middle and lower. The involvement of one or two zones is considered as localized disease, three or four zones as moderate disease, and five or six zones, as extensive disease. Therefore, patients displaying localized or moderate disease were classified as non-severe tuberculosis (nsTB) and those displaying extensive disease and/or cavitations were classified as severe tuberculosis (sTB) [18].

The patients included in this study were distributed into three groups: Group 1: Consisting of 10 patients (8 male and 2 female subjects), aged 22–64 years old, with nsTB bacteriology diagnosis (positive baciloscopy and/or positive culture). Group 2: Consisting of 10 patients (4 male and 6 female subjects), aged 18–65 years old, diagnosed with sTB by bacteriology (positive baciloscopy and/or positive culture). All patients from group 1 and 2 had no history of prior treatment for TB and presented evidences of radiological changes in the thorax. They were treated according to the posological scheme recommended by the Brazilian Health Ministry. Group 3: Consisting of 11 healthy donors (HD) (6 males and 5 females), aged 22–53 years, without previous TB history, without radiological changes in the thorax, and negative tuberculin skin test (TST^−^) (0.1 mL of PPD, in a concentration of 5 units (produced by L*aboratório de Extratos Alergênicos Ltda*, RJ, Brazil and imported from Copenhagen, Denmark). The tests were considered negative for an induration area **<**10 mm measured after 72 h of the inoculation. The clinical evaluation and patients classification were responsibility of SMS.

All subjects included in this evaluation were HIV-negative, with no history of drugs usage and other substance known to affect the immunological status.

### *M. tuberculosis* antigens

*M. tuberculosis*, H37Rv, antigen (MTB-Ag) was provided by the *Micobactérias* Laboratory (Hospital das Clínicas/UFMG/Brazil). The *M. tuberculosis* was cultured in tubes with Loweinstein Jensen medium and incubated at 37°C until evidence of bacterial growth. Colonies were inactivated at 80°C for 1 hour and sonicated in 2 cycles of 20 seconds at 40 Hz in an ice bath. The suspension was then sterilized using gamma radiation (dose of 5000 Gray for 2∶15 hours). The protein concentration was measured by the Lowry method.

### Blood sampling

A peripheral blood sample of 15 mL was collected from each subject using ethylenediamine tetraacetic acid (EDTA) or heparin as anticoagulant. Blood samples from TB patients were collected immediately prior to the beginning of chemotherapy treatment.

### In vitro cultures

PBMC were obtained by separating whole blood over Ficoll (Sigma Chemical Co., St. Louis, MO), stained, and analyzed. Briefly, 1.0×10^7^/mL PBMC were analyzed ex vivo and after being cultured in 96-well plates in 200 µL cultures for 48 h with either medium alone or (MTB-Ag) (10 µg/mL). Brefeldin A (10 µg/mL) was added to the cultures the in the last 4 h of culture. All cultures were carried out using RPMI 1640 supplemented with 5% AB Rh-positive heat-inactivated human serum, antibiotics (200 UI/mL penicillin), and 1 mM L-glutamine (Sigma Chemical Co., St. Louis, MO), in the absence or presence of (MTB-Ag) (10 µg/mL).

### Flow cytometric staining

The immunophenotypic assays of the leukocytes in the peripheral blood were performed according to the protocol at the Center for Infectious Diseases – U.S.A. Briefly, 100 µL aliquots of the whole peripheral blood collected in EDTA were added a cocktail of monoclonal antibodies (mAbs) specific for human lymphocytes cell-surface markers, including anti-CD4- tricolor (TC), CD8-TC, anti-αβ- fluorescein isothiocyanate (FITC), anti-γδ-FITC, anti-CD69- phycoerythrin (PE) and anti-HLADR-PE (BD Biosciences - San Jose, CA, USA). Following incubation, erythrocytes were lysed using 2 mL of FACS lysing solution (Becton Dickinson Biosciences Pharmingen, San Diego, CA, USA), and cells were washed twice with 2 mL of PBS 0.01% of sodium azide. Cell preparations were maintained in 200 µL of FACS fix solution (10 g/L paraformaldehyde, 1% sodium cacodylate, 6.65 g/L sodium chloride) until acquisition in a Becton Dickinson FACS calibur instrument (BD Biosciences - San Jose, CA, USA). At least 35,000-lymphocyte-gated events were acquired for analysis.

To determine intra-cellular cytokine expression pattern, PBMC were incubated with anti CD4-TC, anti CD8-TC, anti αβ- FITC, anti γδ-FITC solutions for 30 min at 4°C. Cells were then washed, fixed, permeabilized with FACS perm buffer (PBS supplemented with 0.5% Bovine Serum Albumin-BSA, 0.5% of saponin and 0.1% sodium azide) and incubated with anti-IFN-γ-PE, anti-TNF-α-PE and anti-IL-10-PE (BD Biosciences - San Jose, CA, USA) solutions for 30 min at 4°C. Then, the preparations maintained in 200 µL of FACS fix solution until acquisition in a Becton Dickinson FACS calibur instrument.

### Data analysis

**Figure 1:**
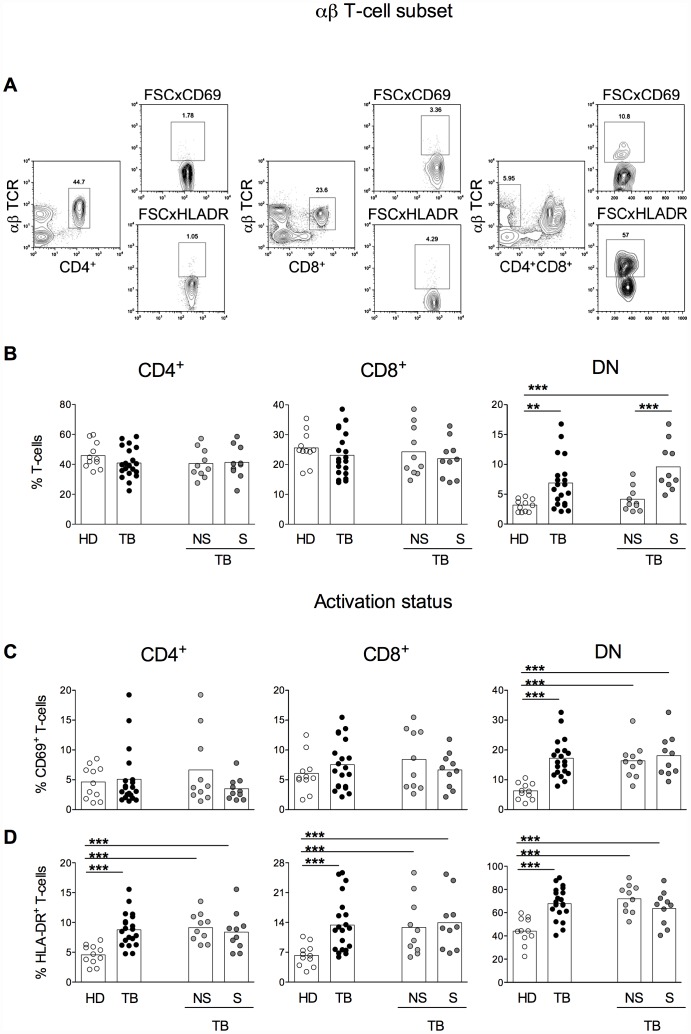
High frequencies of DN αβ T-cells are associated with TB severity. Representative contour plots showing the gate strategy used for the analysis of CD4 (middle left), CD8 (middle center), DN (middle right) αβ-T cells and the expression of CD69 (upper panels) and HLA-DR (lower panels) on DN αβ-T cells (A). Percentages of CD4^+^ (left panels), CD8^+^ (middle panels) and DN (right panels) αβ T-cells in healthy donors (HD, open symbols), TB (total TB, black symbols), nsTB (non-severe TB, light gray symbols) and sTB patients (severe TB, dark gray) were measured before treatment (B). The percentage of CD69 (C) and HLA-DR (D) expression within CD4^+^ (left panels), CD8^+^ (middle panels) and DN (right panels) αβ T-cells in HD, TB, nsTB and sTB patients were analyzed ex vivo. The boxes represent the means.

**Figure 2:**
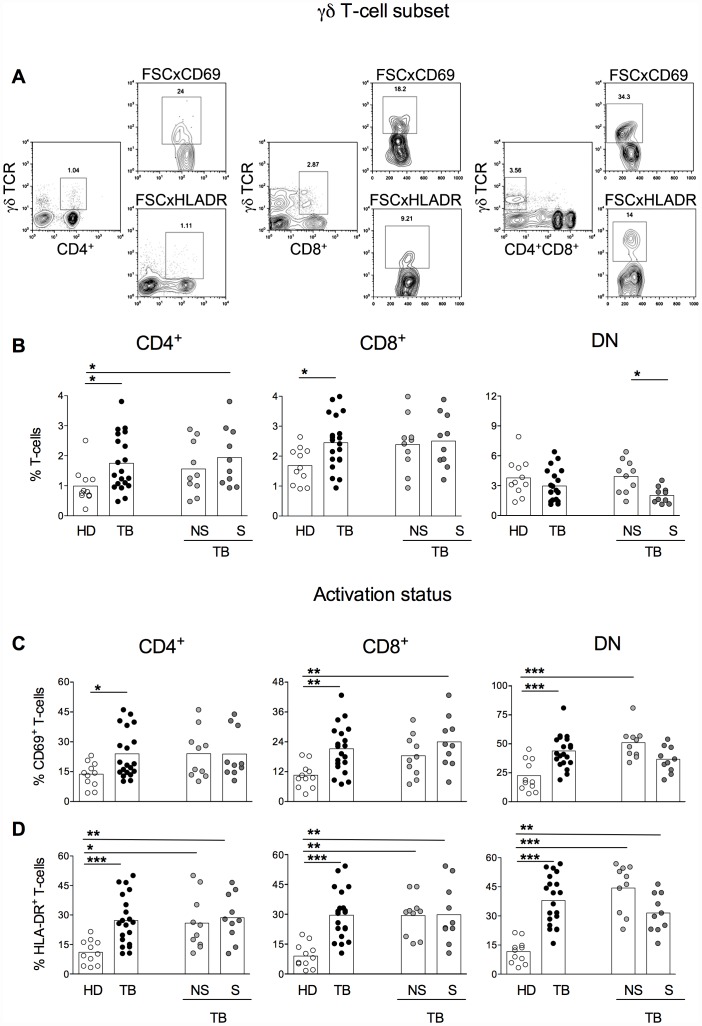
Advanced TB patients display decreased proportions of DN γδ T-cells. Representative contour plots showing the gate strategy used for the analysis of CD4 (middle left), CD8 (middle center), DN (middle right) γδ-T cells and the expression of CD69 (upper panels) and HLA-DR (lower panels) on DN γδ -T cells (A). Percentages of CD4^+^ (left panels), CD8^+^ (middle panels) and DN (right panels) γδ T-cells in healthy donors (HD, open symbols), TB (total TB, black symbols), nsTB (non-severe TB, light gray symbols) and sTB patients (severe TB, dark gray) were measured before treatment (B). The percentage of CD69 (C) and HLA-DR (D) expression within CD4^+^ (left panels), CD8^+^ (middle panels) and DN (right panels) γδ T-cells in HD, TB, nsTB and sTB patients were analyzed ex vivo. The boxes represent the means.

Lymphocytes were analyzed using the software FlowJo 9.3.2. The analysis of lymphocytes subpopulations was accomplished according to distinct gating strategy as briefly described: following lymphocyte gating on size versus granularity, CD4^+^αβ^+^, CD8^+^αβ^+^, CD4^−^CD8^−^αβ^+^, CD4^+^γδ^+^, CD8^+^γδ^+^ and CD4^−^CD8^−^γδ^+^ were selected and evaluated according to their frequencies and the expression of activation the markers CD69 and HLA-DR and the production of the intracellular cytokines IFN-γ, TNF-α and IL-10 (Fig. 1D and 2D).

### Statistics

Statistical analysis was performed comparing HD and TB using Mann Whitney test. For comparisons between HD, nsTB and sTB was performed using Kruskal-Wallis variance analysis followed by Dunn's test for multiple comparisons. Analyses were performed using GraphPad Prism 5.01 software package (San Diego, CA, USA). In all cases, significance was considered at p≤0.05.

## Results

### Higher frequencies of CD4^−^CD8^−^ (DN) αβ T-cells are associated with TB severity

The proportion of CD4^+^, CD8^+^ and CD4^−^CD8^−^ (DN) αβ T-cells, gated as described in Fig. 1A, were analyzed and compared among groups. The frequencies of CD4^+^ and CD8^+^ αβ T-cells were not different between HD and TB patients. Differences were also not observed between the frequencies of CD4^+^ and CD8^+^ αβ T-cells from HD and nsTB or sTB patients, or between nsTB and sTB patients. However, the frequencies of DN αβ T-cells were significantly higher in TB patients than in HD. When the comparison was done between HD and nsTB or sTB subgroups, the difference was seen between HD and sTB patients but not between HD and nsTB patients, indicating that this change happens due the severity of the disease. Corroborating with this finding, sTB patients present higher frequencies of DN αβ T-cells than those classified as nsTB patients (Fig. 1B).

The activation status of different αβ T-cells subsets was analyzed based on CD69 and HLA-DR expression (Fig. 1C). The proportions of CD4^+^ and CD8^+^ αβ T-cells expressing the early activation marker CD69 did not differ among the groups analyzed. However, significantly higher proportions of CD69 expressing DN αβ T-cells were observed in TB patients than in HD. These differences were kept when the frequencies of CD69 expressing DN αβ T-cells were compared between HD and either nsTB or sTB patients.

The expression of HLA-DR was also analyzed (Fig. 1D). The frequencies of HLA-DR expressing CD4^+^, CD8^+^ and DN αβ T-cells were significantly higher in TB patients compared with HD. Differences were also observed in the proportions of HLA-DR expressing CD4^+^, CD8^+^ and DN αβ T-cells between HD and nsTB or sTB. nsTB and sTB displayed similar levels of HLA-DR expression on all αβ T subsets evaluated.

### TB patients with severe pathology display decreased proportions of DN γδ T-cells

The proportion of CD4^+^, CD8^+^ and DN γδ T-cells, gated as described in Fig. 2A, were analyzed and compared among groups. TB patients displayed significantly higher frequencies of CD4^+^ and CD8^+^ γδ T-cells T-cells compared with HD (Fig. 2B). The proportion of CD4^+^ γδ T-cells from sTB patients was by itself higher than the ones observed in HD, however the same was not observed when nsTB and DH individuals were compared. Frequencies of DN γδ T-cells did not differ between total TB patients and HD, but sTB patients displayed lower frequencies of this cell subset when compared with nsTB patients. Thus, lower frequencies of DN γδ T-cells might suggest a severe form of tuberculosis.

Distinct of the αβ T-cells, the frequencies of CD69 expressing cells were higher on CD4^+^, CD8^+^ and DN γδ T-cells from TB patients compared with HD (Fig. 2C). When the CD69 expression was analyzed in CD8^+^ γδ T-cells, its expression was also higher in sTB patients the compared with HD. The same did not hold true for CD4^+^ and DN γδ T-cell populations. Moreover, the opposite was seen for the DN γδ T-cell subset. The increased frequencies of CD69 expressing cells in TB patients were due the high expression observed in the nsTB patients group compared to HD.

The frequencies of HLA-DR expressing cells were also analyzed on CD4^+^, CD8^+^ and DN γδ T-cells (Fig. 2D). The frequencies of HLA-DR expressing cells were significantly higher in TB patients compared with HD in the CD4^+^, CD8^+^ and DN γδ T-cell subsets. Differences were also observed in the proportions of HLA-DR expressing CD4^+^, CD8^+^ and DN γδ T-cells between HD and nsTB or sTB. No differences were observed in HLA-DR expression on all the γδ T subsets evaluated when nsTB and sTB were compared.

### Higher frequencies of IFN-γ producing DN αβ T-cells were found in nsTB patients

**Figure 3:**
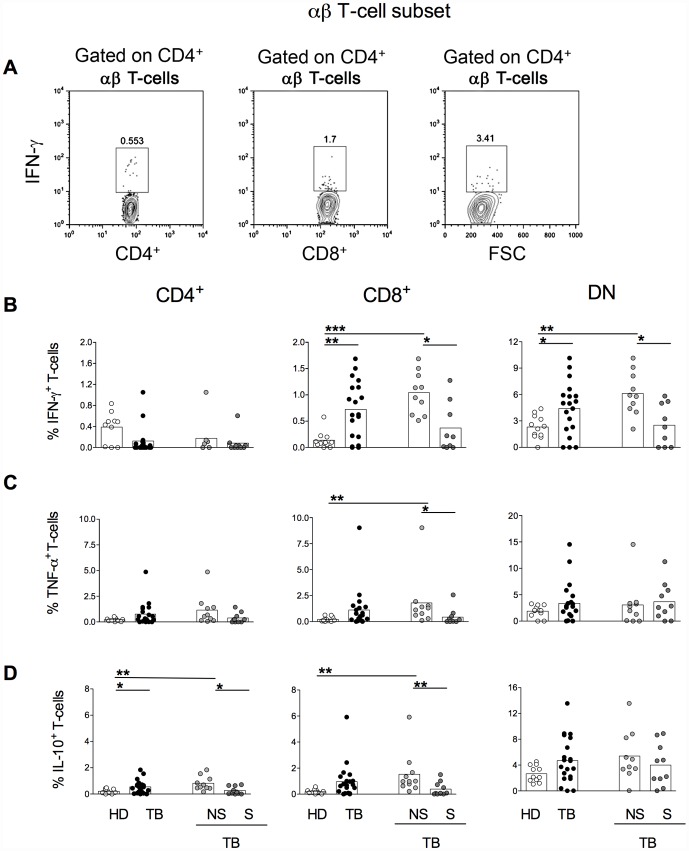
Higher frequencies of IFN-γ producing DN αβ T-cells are found in nsTB patients. Representative contour plots showing the proportions of IFN-γ producing CD4 (left panel), CD8 (middle panel) and DN (right panel) αβ-T cells (A). The percentages of IFN-γ (B), TNF-α (C) and IL-10 (D) expression within CD4^+^ (left panels), CD8^+^ (middle panels) and DN (right panels) αβ T-cells in healthy donors (HD, open symbols), TB (total TB, black symbols), nsTB (non-severe TB, light gray symbols) and sTB patients (severe TB, dark gray) were measured before treatment. PBMCs were stimulated with (MTB-Ag) for 48 hours. The boxes represent the means.

Since distinct groups of TB patients displayed different proportions of T-cell subsets and their activation status, we next evaluated the ability of each T-cell population to produce inflammatory and modulatory cytokine upon in vitro (MTB-Ag)-specific stimulation (Fig. 3). Frequencies of IFN-γ producing CD4^+^ αβ T-cells did not differ significantly among all the groups analyzed (Fig. 3B). However, higher frequencies of IFN-γ producing CD8^+^ and DN αβ T-cells were seen in TB patients than in HD. The differences observed in the proportions of IFN-γ producing cells between TB and HD individuals were probably caused by the patients presenting the non-severe TB, since nsTB patients presented much higher frequencies of IFN-γ producing CD8^+^ and DN αβ T-cells than either HD or sTB patients. It is important to mention that in CD8^+^ cells displayed higher frequencies of IFN-γ producing cells compared with CD4^+^ cells from TB patients.

Differences in TNF-α producing cells were only seen in the CD8^+^ αβ T-cell subset. nsTB patients displayed higher frequencies of TNF-α producing CD8^+^ αβ T-cells than HD (Fig. 3C). As observed for IFN-γ, the frequencies of TNF-α producing cells were significantly higher in nsTB patients when compared with sTB ones.

Higher frequencies of the IL-10 producing CD4^+^ αβ T-cells were found in TB patient compared with HD (Fig. 3D). Differences became even higher when the frequencies of IL-10 producing CD4^+^ αβ T-cells were compared between nsTB and HD. Moreover, between the TB groups, nsTB displayed higher proportion of IL-10 producing CD4^+^ αβ T-cells than sTB. The same was observed for the CD8^+^ αβ T-cell subset. nsTB displayed higher proportion of IL-10 producing CD8^+^ αβ T-cells than sTB. And differences in the frequencies of IL-10 producing CD8^+^ αβ T-cells were only between nsTB and HD individuals. Together these findings indicate that both inflammatory and modulatory cytokine production is suppressed in TB patients presenting the more severe clinical presentation of the disease.

### DN γδ T-cells from nsTB patients produce inflammatory cytokines whereas sTB produce IL-10

**Figure 4:**
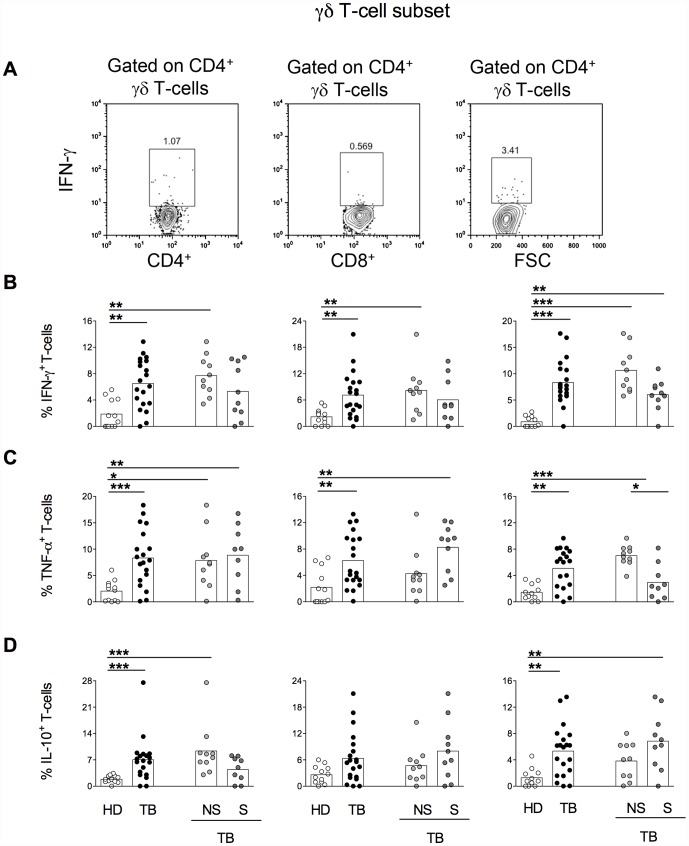
DN γδ T-cells from nsTB patients produce inflammatory cytokines whereas those from sTB produce more IL-10. Representative contour plots showing the proportions of IFN-γ producing CD4 (left panel), CD8 (middle panel) and DN (right panel) γδ-T cells (A). Percentages of IFN-γ (B), TNF-α (C) and IL-10 (D) within CD4^+^ (left panels), CD8^+^ (middle panels) and DN (right panels) γδ T-cells in healthy donors (HD, open symbols), TB (total TB, black symbols), nsTB (non-severe TB, light gray symbols) and sTB patients (severe TB, dark gray) were measured before treatment. PBMCs were stimulated with (MTB-Ag) for 48 hours. The boxes represent the means.

Higher frequencies of IFN-γ producing CD4^+^, CD8^+^ and DN γδ T-cells were found in TB patients when compared with HD (Fig. 4B). These differences were maintained when the subgroup nsTB patients was compared with HD. Thus, higher proportions of IFN-γ producing cells were observed within CD4^+^, CD8^+^ and DN γδ T-cells.

As for IFN-γ, differences in TNF-α producing CD4^+^ γδ T-cells were seen between TB patients and HD (Fig. 4C). However, both nsTB and sTB patients displayed similar higher frequencies of TNF-α producing CD4^+^ γδ T-cells than HD. Proportion of TNF-α producing CD8^+^ γδ T was also higher in total TB and sTB patients than in HD. Similarly to the others γδ T-cell subsets, TNF-α producing DN cells were more frequent in TB patients than HD. nsTB also displayed higher proportion of TNF-α producing DN γδ T-cells when compared with HD. Only among the DN γδ T-cells, nsTB patients displayed higher frequencies of TNF-α producing cells when compared with patients presenting the more severe form of the disease.

TB patients also presented higher frequencies of IL-10 producing CD4^+^ and DN γδ T-cells when compared with HD (Fig. 4D). Considering the CD4^+^ γδ T-cell subpopulation, the nsTB group was the responsible for this difference; on the contrary for the DN γδ T-cells the sTB patients were the ones responsible for the increased frequencies of IL-10 producing cells.

## Discussion

The complexity of tuberculosis is created through the interaction between a range of mycobacteria strains with a heterogenic host immune response. Despite the complex range of diseases and responses associated with them, several cytokines and their cellular sources have been correlated with the cure for and/or pathology of tuberculosis.

In this report, we establish that the DN lymphocyte population from *M. tuberculosis*-infected patients is composed of αβ and γδ DN T-cells that express a more pronounced activated and inflammatory profile compared to DN T-cells from non-infected individuals. While the proportions of CD4^+^ and CD8^+^ αβ T-cells do not alter upon infection, the proportions of DN αβ T-cells are higher in TB-infected patients than in healthy donors. Moreover, higher frequencies of DN αβ T-cells are found in patients presenting the severe form of the disease when compared to those presenting the non-severe form. DN αβ T cells display a restricted TCR repertoire that recognizes some bacterial antigens in the context of the MHC class 1b molecules and high bacillary load would leads to the expansion of these antigen-specific T cell subpopulations in severe TB [19], [20]. On the other hand, proportions of γδ DN T-cells are not different between healthy donors and TB-infected patients when they were analyzed as a whole; however, differences are found between patients presenting the severe and non-severe form of the disease. Frequencies of γδ T-cells were reported before, and were significantly greater in patients with protective and resistant immunity, defined by the authors as tuberculin reactors, than in those with ineffective immunity [21]. Despite αβ and γδ DN T-cells are present in a relative minority compared to other T-cell populations, their highly activated profile makes they likely important in the overall immune response against *M. tuberculosis* as was previously suggested [9], [22].

Up to date there are no sufficiently validated biomarkers to aid the evaluation of new tuberculosis vaccine candidates, the improvement of tuberculosis diagnostics or the development of more effective and shorter treatment regimens [23]. Furthermore, host biomarkers in tuberculosis are needed to provide correlates of risk, protection, and response to therapy. In the present study, αβ and γδ DN T-cells from infected patients expressed increased levels not only of CD69 but also higher frequencies of HLA-DR expressing cells ex vivo, which are indicators of recent antigenic exposure. Increased expression of HLA-DR in patients with TB was reported before, but no correlation with clinical outcome was done [24]. The expression of either CD69 or HLA-DR on αβ DN T-cells of infected patients is similarly increased in TB patients presenting the non-severe and severe form of the disease. γδ DN T-cells from TB patients also display an activated phenotype compared with healthy donors. Thus, overall, the DN T population from TB-infected patients presented a profile compatible with previous antigen exposure (HLA-DR) and recent activation (CD69).

Host effector immune response against *M. tuberculosis* is related to the presence of a strong Th1 response and memory, leading to the production of immune mediators that activate parasite-infected macrophages for parasite destruction. One critical cytokine for host control of *M. tuberculosis* is IFN-γ. IFN-γ is required for induction of NO synthase type 2 and other effector molecules in infected macrophages. Both CD4^+^ and CD8^+^ T-cells and NK cells have been shown to be sources of this protective cytokine in *M. tuberculosis* infection [13], [16]. The essential role of IFN-γ is evident from the increased risk of tuberculosis in: (i) individuals with deficiency of IFN-γ and interleukin-12, which promotes Th1 cell differentiation; (ii) animal models depleted of CD4^+^ T-cell during the experimental infection; (iii) HIV-infected individuals [25], [26]. αβ DN T-cells from TB-patients displayed a higher commitment to the production of IFN-γ. Moreover they also contained a higher proportion of IFN-γ producing cells than the CD4^+^ and CD8^+^ αβ T-cell population. High frequencies of IFN-γ producing cells in TB group are accounted by patients presenting the non-severe form of tuberculosis. A great proportion of αβ DN T-cells from nsTB patients are maintain the ability of IFN-γ production, which is lost for sTB patients. The reduction of TB-specific T-cells and the impairment of Th1 immune response in active pulmonary tuberculosis patients were reported before [27], [28].

γδ DN T-cells not only presented higher frequencies of IFN-γ producing cells, but they also contained a higher frequency of cells producing another inflammatory cytokine, TNF-α. Besides IFN-γ, TNF-α is also a key molecule in host immunity to tuberculosis. The lack of this cytokine leads to reduced expression of immune mediators and increased susceptibility to primary infection with *M. tuberculosis*, and depletion of TNF after infection results in reactivation of latent disease [29], [30], [31], [32]. Despite studies have failed to control *M. tuberculosis* in human host cells in vitro, its role in vivo is clearly shown by the reactivation of latent disease upon anti–TNF treatment [33], [34], [35]. The high commitment of DN T-cells to cytokines known to be effector mediators in controlling mycobacterium suggests their participation in the immune responses during this disease.

Higher frequencies of CD4^+^ and CD8^+^ αβ T-cells producing the modulatory cytokine IL-10 were found in TB-infected patients. In fact, studies have been demonstrated that newly diagnosed patients, before treatment produce high levels of IL-10 and low amounts of IL-12, while the reverse was true in healthy controls and successfully treated patients [36]. IL-10 suppresses macrophage functions, including killing of intracellular pathogens and TNF and IL-12 production required for Th1 responses [37], [38]. Due to its regulatory profile, it is likely that IL-10 induction during tuberculosis will affect the course of disease. IL-10 message is induced during experimental infection with a number of mycobacterial species, and has been correlated with enhanced disease in TB patients [39], [40]. Moreover, in an animal model of tuberculosis, the deficiency of IL-10 reduced bacterial load in lungs with decreased dissemination to the spleen, which was preceded by an earlier and enhanced Th1-type response [41]. Interestingly, DN αβ T-cells from TB-infected patients do not produce more IL-10 than the same subset from healthy donors, in opposed to higher frequencies of IFN-γ found in DN αβ T-cells from these patients. The opposite is observed in CD4^+^ αβ T-cells subset, where no differences were found in IFN-γ production among groups, but IL-10 producing cells were prominent among CD4^+^ αβ T-cells from TB patients, especially those presenting the non-severe form of the disease. This was an interesting finding, and might explain in part the fact that DN αβ T-cells are able to maintain for longer their ability to produce inflammatory cytokines in patients presenting the non-severe form of the disease. On the contrary, higher frequencies of IL-10 producing cells were found in γδ DN T-cells from TB-infected patients, due to the severe form of tuberculosis, which together with the lower IFN-γ production suggest a modulatory role of γδ DN T-cells during tuberculosis. Although it has been shown that the γδ T-cells are expanded within PBMC from patients presenting this disease upon stimulation in vitro and from health care workers who were tuberculin skin test positive and who had constant contact with patients with active tuberculosis, the precise role of this subpopulation in tuberculosis is still not clear [21], [42].

Thus, in TB-infected patients, the inflammatory components that reside within the αβ and γδ DN T-cell subpopulations are maintained among patients presenting the non-severe form of the disease, while the modulatory component within γδ DN T-cells takes place in more advanced forms of tuberculosis. The inflammatory profile in nsTB patients will favor the activity of DN T-cells as inducers of cell-mediated immunity through the activation of phagocytes and the induction of Th1 differentiation. Overall, our findings suggest that the cytokine profile of αβ and γδ DN T cells seems to contribute to the development/maintenance of distinct clinical aspects of TB, as part of the complex immunological network triggered by the TB infection.
